# Magnetically Induced Transparency in Media with Helical Dichroic Structure

**DOI:** 10.3390/ma14092172

**Published:** 2021-04-23

**Authors:** Ashot H. Gevorgyan, Sergey S. Golik, Nikolay A. Vanyushkin, Ilya M. Efimov, Mushegh S. Rafayelyan, Hermine Gharagulyan, Tatevik M. Sarukhanyan, Meruzhan Z. Hautyunyan, Gvidon K. Matinyan

**Affiliations:** 1 School of Natural Sciences, Far Eastern Federal University, 10 Ajax Bay, Russky Island, 690922 Vladivostok, Russia; golik_s@mail.ru (S.S.G.); vaniuschkin.nick@yandex.ru (N.A.V.); efimov.im@dvfu.ru (I.M.E.); 2 Institute of Automation and Control Processes, Far East Branch, Russian Academy of Sciences, 690041 Vladivostok, Russia; 3 Department of Physics, Yerevan State University, 1 Alex Manukyan Str., 0025 Yerevan, Armenia; mrafayelyan@gmail.com (M.S.R.); s.tatevik93@gmail.com (T.M.S.); meruzhzh@gmail.com (M.Z.H.); 4 Institute of Chemical Physics NAS RA, 5/2, P. Sevak Str., 0014 Yerevan, Armenia; hermghar@gmail.com; 5 Department of Agrarian Engineering, Armenian National Agrarian University, 74, Terian Str., 0009 Yerevan, Armenia; matinyang@yandex.ru

**Keywords:** magneto-optical activity, cholesteric liquid crystal, magnetically induced transparency, eigen polarization, spectra of reflection, spectra of transmission, spectra of absorption

## Abstract

In our paper, the magneto-optical properties of a dichroic cholesteric liquid crystal layer with large values of magneto-optical parameter g and low values of dielectric permittivity were investigated. The solutions of the dispersion equation and their peculiarities were investigated in detail. The specific properties of reflection, transmission, absorption, rotation, ellipticity spectra and also the spectra of ellipticity and azimuth of eigen polarization were investigated. The existence of a tunable linear and nonreciprocal transmission band was shown.

## 1. Introduction

Helical structures, artificially created or self-organizing, are present everywhere and their investigation is still ongoing. They have attractive optical properties. Cholesteric liquid crystals (CLCs) are formed from long molecules that are attached to dissolved chiral molecules in such a way that they form a helicoidal structure [1,2,3,4]. The periodic structure of a CLC leads to the appearance of a polarization-sensitive photonic band gap (PBG). The spatial period (helix pitch) of the CLC is in the optical region and it, as well as the local optical axis, can easily change under the influence of various physical factors [3,4,5,6,7]: pressure, temperature, electric and magnetic fields, being under the influence of impurities and interfaces. The amenability of CLCs, and of liquid crystals in general, to external influences is responsible for their wide appeal.

In this work, we investigated the optical properties of CLCs that exhibit optical activity in the presence of an external static magnetic field. The magneto-optical properties of CLCs and chirally sculptured thin films were considered in [8,9,10,11,12], and this topic remains relevant [13]. On the other hand, as is well known, due to quantum interference an electromagnetically induced transparency (EIT) can be observed in atomic physics, which results in a narrowband transparency window for originally opaque medium [14]. Later, this concept was extended to classical optical systems using gas-phase atomic [15,16], metamaterial/metasurface [17,18], plasmonic [19,20], optical [21,22,23,24,25,26], optomechanical [27,28] and superconducting [29,30] systems, which allow experimental implementation of incoherent light and operation at room temperature [31,32]. A new effect, namely the effect of magnetically induced transparency, was discovered in [33], which is observed in magnetically active helically structured periodical medium. In this paper, we investigated specific properties of magnetically induced transparency in media with a helical structure in the absence of local birefringence. Of course, a CLC structure intrinsically needs the existence of local birefringence for the helix to be defined. However, in this paper, we consider the CLC without local birefringence in order to clearly distinguish the effects arising from the material helical dichroism. Moreover, an external magnetic field can also directly affect the local dielectric tensor components, being a new mechanism of local dielectric anisotropy, but as it is a quadratic in the external field effect, we can initially neglect it. Then, the external magnetic field distorts the structure of the CLC, but since we consider the case of CLC without a local dielectric or magnetic anisotropy we can neglect this influence as well. On the other hand, the problem in this formulation (consideration of the limiting case ReΔ→0, where ReΔ=Reε1−Reε22, and Reε1 and Reε2 are the real parts of the principal values of the CLC local dielectric tensor) allows us to reveal interesting new manifestations of this effect, as well as provides a more complete understanding of some of the features of CLC optics. It allows us to observe the simultaneous presence of diffraction transmission (not reflection) and magnetically induced transmission. In our future works we plan to continue our investigations towards more natural configurations of CLC materials with local birefringence and magneto-optical activity. Alternatively, at the end of the next section, we will consider the case of a CLC with local dielectric anisotropy, and we will take into account the influence of external magnetic field on the CLC helix pitch and on the principal values of the dielectric tensor components.

## 2. Models and Methodology

**Figure 1:**
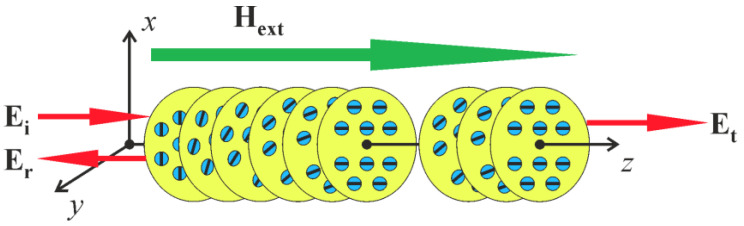
The geometry of the problem. The large yellow circles represent the *xy* cross-sections of magneto active CLC along the *z*-axis. They are embedded with small blue circles which are the cross-sections of the isotropic molecules without birefringence. The black lines in these circles correspond to the directions of the absorption oscillators, these directions are continuously changing forming a helicoidal structure along the z-axis.

The dielectric permittivity and magnetic permeability tensors of a magneto-active CLC (Figure 1) have the forms:

(1) $$\hat{\varepsilon }\left(z\right)=\varepsilon_{m}\left(\begin{array}{ccc} 1+\delta \cos2az & \pm \delta \sin2az\pm ig/\varepsilon_{m} & 0\\ \pm \delta \sin2az\mp ig/\varepsilon_{m} & 1-\delta \cos2az & 0\\ 0 & 0 & 1-\delta \end{array}\right),\;\hat{\mu }\left(z\right)=\hat{I},$$

where $\varepsilon_{m}=\left(\varepsilon_{1}+\varepsilon_{2}\right)/2$, $\delta =\frac{\left(\varepsilon_{1}-\varepsilon_{2}\right)}{\left(\varepsilon_{1}+\varepsilon_{2}\right)}$, $\varepsilon_{1,2}=\varepsilon_{0}+i\varepsilon_{1,2}^{\prime \prime }$, $\varepsilon_{0}$ is the real part of the dielectric tensor components, which is assumed to be the same for all components, g is the parameter of magnetooptical activity, a=2π/p, p is the helix pitch installed in an external magnetic field. As mentioned above, the external magnetic field can lead not only to the Faraday effect, but it can also directly affect the Re$\varepsilon_{1,2}$ values with a quadratic scaling, see, in particular [34]. Below, as the first step we neglect the changes of Re$\varepsilon_{1,2}$. We consider the light propagation towards the direction of the helix axis without accounting the consequences of optical dispersion or absorption dispersion, i.e., the components of the dielectric and magnetic tensors are constant having no dependencies on frequency; and the same applies for the imaginary parts as well.

Using the method of converting the wave equation to the field components relative to the *x*′ and *y*′ axes, which rotate jointly with the structure [35,36] (the axis x′ is oriented toward the local optical axis everywhere, while the y′ axis is perpendicular to the axis x′), we derive the following dispersion relation:

(2) $$k_{m}^{4}+a_{1}k_{m}^{2}+a_{2}k_{m}+a_{3}=0$$

where $k_{m}$ is the *z* component of the *m*th wave vector in the rotating frame (m=1,2,3,4), $a_{1}=-2\left(\frac{\omega^{2}}{c^{2}}\varepsilon_{m}+a^{2}\right)$, $a_{2}=-4\frac{\omega^{2}}{c^{2}}ag,$ $a_{3}=-2\frac{\omega^{2}}{c^{2}}a^{2}\varepsilon_{m}+\frac{\omega^{4}}{c^{4}}\varepsilon_{m}^{2}\left(1-\delta^{2}\right)-\frac{\omega^{4}}{c^{4}}g^{2}+a^{4}$, ω is angular frequency and *c* is light speed in a vacuum. Based on the dispersion equation, we can pass to the problem of finding the reflection, transmission, and light localization for the magnetoactive CLC layer of finite thickness. Let us note that our simulation is based on an imaginary system with thickness *d* surrounded by isotropic spaces. In real systems, the boundary conditions are considerably complicated because of the anchoring energies on the solid substrate which provide a dynamic evolution of the tilt angle with the applied field. Then, we assume the CLC helix is perpendicular to the layer boundaries and directed along the *z*-axis. The CLC layers on both sides are surrounded by isotropic half-spaces with the same refractive index (*n_s_*). The boundary conditions are based on the continuity of tangential components of the electric and magnetic fields and can be represented as a system of eight linear equations with eight unknowns (for more details see [37]). By solving this boundary-value problem, one can obtain the components of the reflected $E_{r}\left(z\right)$ and transmitted $E_{t}\left(z\right)$ fields, as well as for the field $E_{in}\left(z\right)$ inside the CLC layer itself and, thus, is able to the reflection R=|Er|2|Ei|2, transmission T=|Et|2|Ei|2, and absorption A=1−(R+T) coefficients, rotation of the plane of polarization (3) and polarization ellipticity of transmitted light (4), and photonic density of state (PDS) (5):

(3) φ=12arctg(2Reχ1−|χ|2),

(4) e=tg(12arcsin(2Imχ1+|χ|2)),

(5) $$\rho_{m}=\frac{dk_{m}}{d\omega }=\frac{1}{d}\frac{\frac{du_{m}}{d\omega }\nu_{m}-\frac{d\nu_{m}}{d\omega }u_{m}}{u_{m}^{2}+\nu_{m}^{2}},$$

where *d* is the thickness of the CLC layer, and *u_m_* and *v_m_* are the real and imaginary parts of the amplitude of the transmitted wave, χ=EtyEtx, $E_{i}$ is the field of the incident wave, $E_{t}^{x,y}$ are the *x* and *y* components of the transmitted wave, and the values *m* = 1, 2 correspond to the diffracting and non-diffracting eigenmodes of the CLC layer, respectively. The PDS for an isotropic layer with a refractive index *n* has the form: $\rho_{iso}=n/c$, where *c* is the speed of light in a vacuum. Further, below, all calculations were made for a magneto-active CLC layer with the following parameters: $\varepsilon_{0}=0.5$, the CLC layer helix is right-handed, its pitch is *p* = 400 nm, and the CLC layer thickness is *d* = 5*p*. To minimize the influence of dielectric borders, we consider the case $n_{s}=\sqrt{\varepsilon_{m}}$, which is when the CLC layer is sandwiched between two half-infinite isotropic spaces with the same refractive index, which equals to the CLC layer average refractive index.

## 3. Results and Discussion

In this section, we will present the results of our simulations. Solving the dispersion equation (Equation (2)), we get the wavelength dependences of the wave vectors in the medium. Then, solving the boundary value problem, we pass to the presentation of spectra of reflection, transmission, rotation of the plane of polarization and polarization ellipticity, PDS, azimuth and ellipticity of the EP, as well as the evolution of reflection, transmission, and absorption spectra with a change in the magneto-optical parameter g. At the end of this section, we will present the results of studying the peculiarities of light localization by presenting the dependences of the intensity of the total wave excited in the medium layer, the evolution of the localized energy density with a change in the magneto-optical activity parameter g, and the dependence of the integral energy in a finite spectral range, again on parameter g.

**Figure 2:**
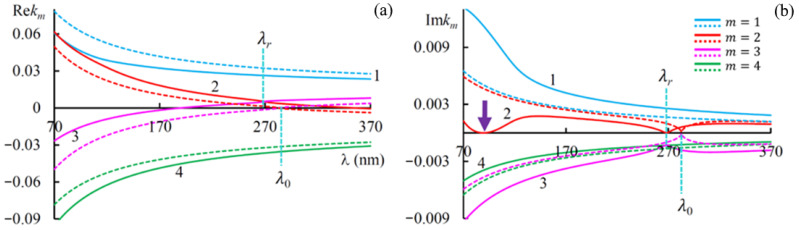
The dependences of Rekm (**a**) and Imkm (**b**) on the wavelength *λ* in the case of g=0.3 (solid lines) and g=0 (dashed lines). The imaginary parts of the principal values of local dielectric tensors are as follows: Imε1=0.2, Imε2=0.

Figure 2 shows the dependences of Rekm and Imkm on the wavelength *λ* at different values of g. As seen in Figure 2, in the absence of an external magnetic field, the curves Re$k_{mz}\left(\lambda \right)$ and Im$k_{mz}\left(\lambda \right)$ are symmetric about the axis $k_{mz}=0$, which can be explained by the reciprocity of the system when an external magnetic field is absent. In addition, we have a wavelength, $\lambda_{0}=p\sqrt{\varepsilon_{0}}=282.8$ nm, where two of the four wave numbers equal zero. They are resonance wave numbers in the absence of local refraction. The other two wave numbers are the propagating modes, and we will call these wave numbers non resonant.

We enumerated the eigen solutions of Equation (1) for the non-resonance and the resonance wave vectors by *m* = 1 and 4, and *m* = 2 and 3, respectively.

The presence of an external magnetic field causes asymmetric displacement of both the curves, Rekm(λ) and Imkm(λ), with regard to the axis $k_{m}=0$, and nonreciprocity appears. As one can see in Figure 2a, in the case g > 0 (g = 0.3), the real parts of the curves Rek1,4(λ) are displaced downwards, and ones of the curves Rek2,3(λ) are displaced upwards, for the imaginary parts Imk1,4(λ) and Imk2,3(λ) we have the opposite situation (see Figure 2b). We also note that the wavelength *λ_r_* at which the curves of the real parts of the resonance wave numbers Rek2(λ) and Rek3(λ) intersect is shifted towards the short waves with respect to *λ*_0_. At this wavelength Imk2=0 and Imk3 take on their maximal values. Let us note one more peculiarity: as can be seen from Figure 2b, at a certain wavelength (at $\lambda_{t}=89.4$ nm for the given problem parameters), the value Im$k_{2z}$ equals zero, that at this wavelength medium is transparent despite Imε1≠0.

**Figure 3:**
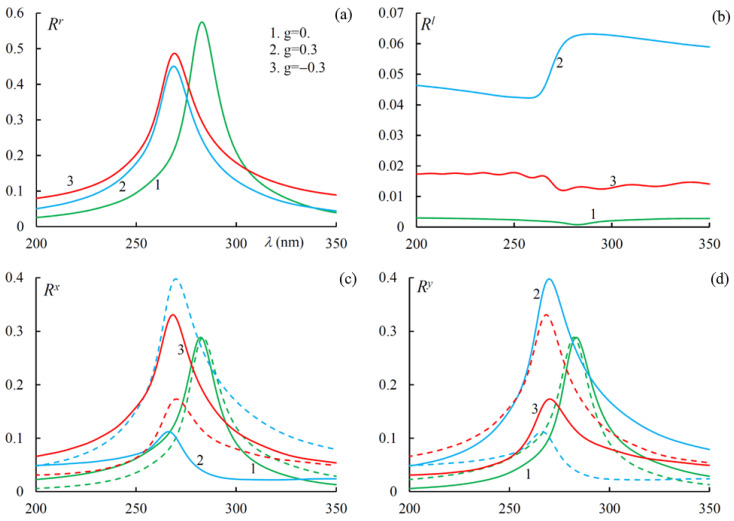
The reflection spectra at different values of g. The incident light has (**a**) right circular, (**b**) left circular, (**c**) linear along the x axis, and (**d**) linear along the y axis polarizations. Solid lines correspond to Imε1=0.2, Imε2=0, and dashed lines to Imε1=0.2, Imε2=0.

Now we address the reflection, transmission, and absorption peculiarities of a CLC layer of finite thickness. Figure 3 shows the reflection spectra for different incident light polarizations at various values of g. As seen in Figure 3, PBGs exhibit a blueshift upon increasing the parameter g. Furthermore, as these spectra show, the reflection coefficients for incident light with right and left circular polarizations are the same in these two cases, while they are different for orthogonal linear polarizations, which was expected.

**Figure 4:**
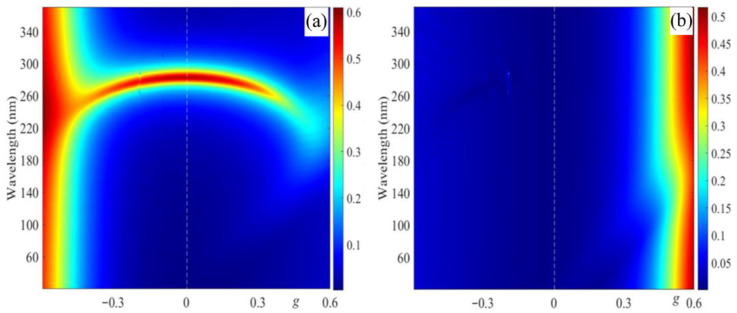
The evolution of the reflection spectra with a change in g for incident light with polarization coinciding with its (**a**) diffracting EP and (**b**) non-diffracting EP.

Figure 4 shows the reflection spectra dependence on parameter g for incident light polarizations coinciding with eigen polarizations (EPs). The incident light with EPs is transmitted through the system without any change in the state of polarization. The EPs coincide with the polarizations of the eigenmodes. In specific cases, the EPs of the medium coincides approximately with orthogonal circular polarization for the normal incident light, although generally they can vary significantly from the circular polarizations. We assume the first EP is the one diffracting on the periodic structure (at g = 0), i.e., it almost coincides with the right-hand circular polarization.

As is seen in Figure 4, the PBG for the first (diffracting) EP is shifted to shorter wavelengths upon increasing parameter g (modulus), as also mentioned above. Nonreciprocal reflection takes place (the reflection spectra are non-symmetric with regard to axis g = 0 (white dashed line)). As one can see from these results, such a structure can be used as a broadband optical diode, provided that the condition g > ε_0_ is met.

**Figure 5:**
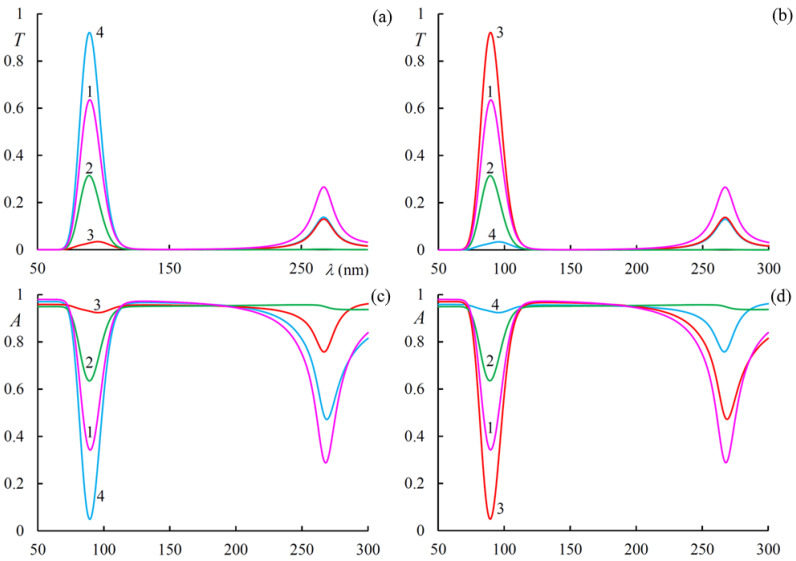
The transmission (**a**,**b**) and absorption (**c**,**d**) spectra at g = 0.3 for (**a**,**c**) Imε1=0.2, Imε2=0., and (**b**,**d**) Imε1=0., Imε2=0.2. Curves 1, 2, 3, and 4 are enumerated according to the right circular, left circular, linear along the x axis, and linear along y axis polarizations, correspondingly.

Figure 5 shows the transmission and absorption spectra for different incident light polarizations at g = 0.3. First of all, we want to note that at wavelength *λ_r_* for incident light with right-hand circular polarization, both diffraction reflection (the reflection coefficient) and diffraction transmission (the transmission coefficient) have a maximum, and the absorption has a minimum here. The same thing takes place at wavelength *λ*_0_ at g = 0. Further, at wavelength *λ_t_*, we almost have complete transmission (and, accordingly, practically no absorption) for incident light that is linear along *x* axis polarization at Imε1=0.2, Imε2=0, and, accordingly, for incident light that is linear along *y* axis polarization at Imε1=0, Imε2=0.2. This is the effect of magnetically induced transparency; and is observed only in the presence of external magnetic field. This effect is nonreciprocal, that is, it does not express in the case of g < 0. With an increase in external magnetic field strength (with increasing parameter g), this peak of transparency shifts to larger wavelengths. This transparency window is controllable. As with reflection, we have the same transmission (absorption) both at Imε1=0.2, Imε2=0, and at Imε1=0, Imε2=0.2 for orthogonal circular polarizations.

**Figure 6:**
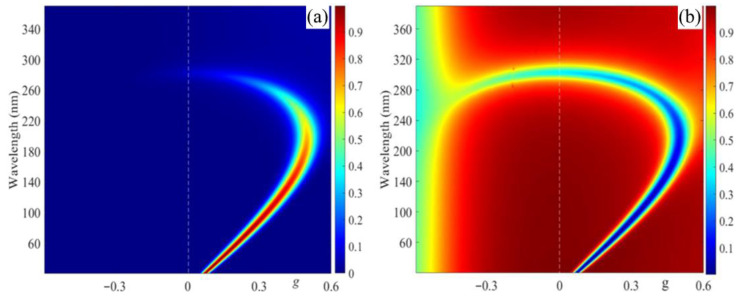
The evolution of the transmission (**a**) and absorption (**b**) spectra with a change in parameter g for incident light with polarization coinciding with its diffracting EP.

Figure 6 shows the evolution of the transmission and absorption spectra with a change in parameter g for incident light with polarization, coinciding with its diffracting EP. As is seen in Figure 6, with an increase in parameter g, the magnetically induced transparency band undergoes a redshift, while the diffraction transmission band undergoes a blue shift. At a certain value of g, they merge into a single band.

**Figure 7:**
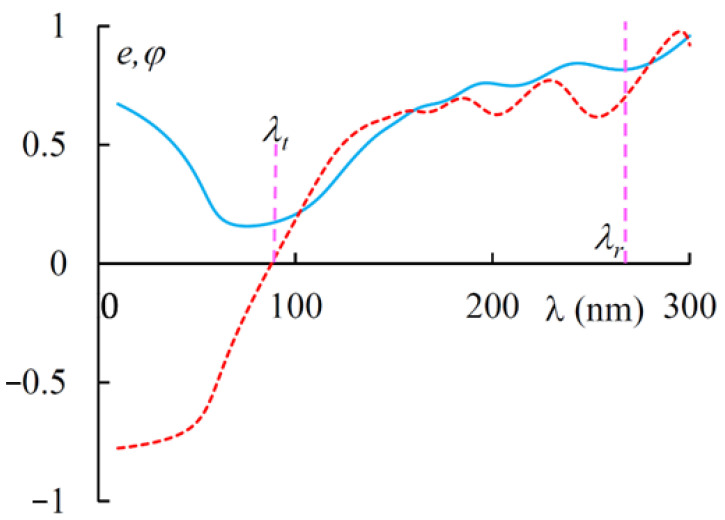
The spectra of ellipticity *e* (solid line) and polarization.

Figure 7 shows the spectra of ellipticity *e* and polarization plane rotation *φ*. As is seen in Figure 7, the rotation makes sharp changes near the magnetically induced transparency band and changes its sign when passing through this band. The ellipticity of transmitted light (at the incidence on the layer of linearly polarized light) has its minimum again in this magnetically induced transparency band.

**Figure 8:**
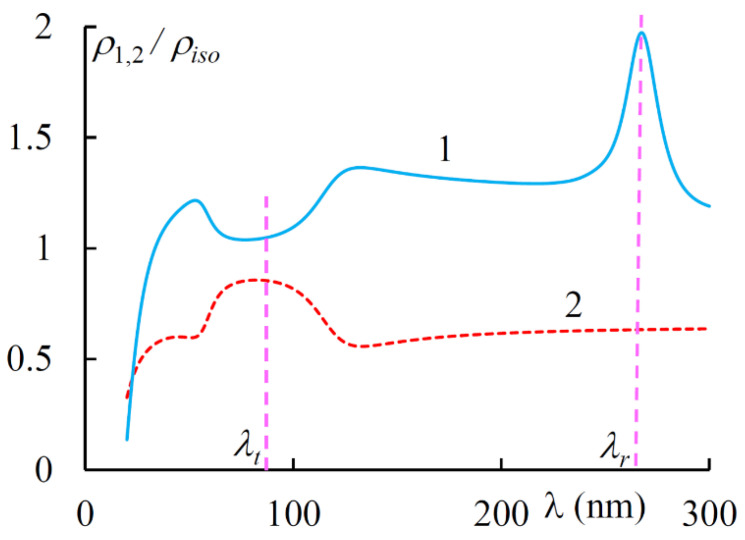
The spectra of PDS for the two EPs at g = 0.3.

**Figure 9:**
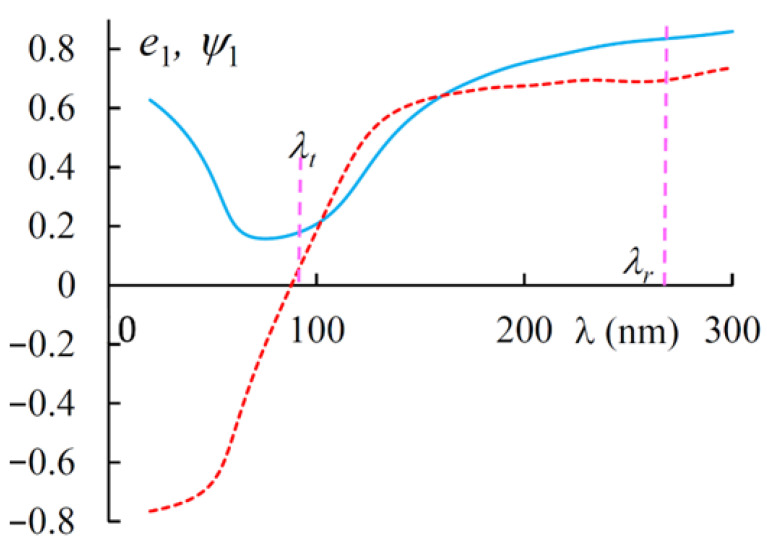
The spectra of ellipticity $e_{1}$ (solid line) and azimuth $\psi_{1}$ (dashed line) of the diffracting EP at g = 0.3.

Figure 8 shows the spectra of PDS for diffracting and non-diffracting EPs. Figure 9 shows the spectra of ellipticity $e_{1}$ and azimuth $\psi_{1}$ of the first EP. For the second EP, we have $e_{2}=-e_{1}$ and $\psi_{2}=-\psi_{1}$. As is seen in Figure 9 the EPs near the transparency band are quasi linear, while, in general, they are quasi circular.

**Figure 10:**
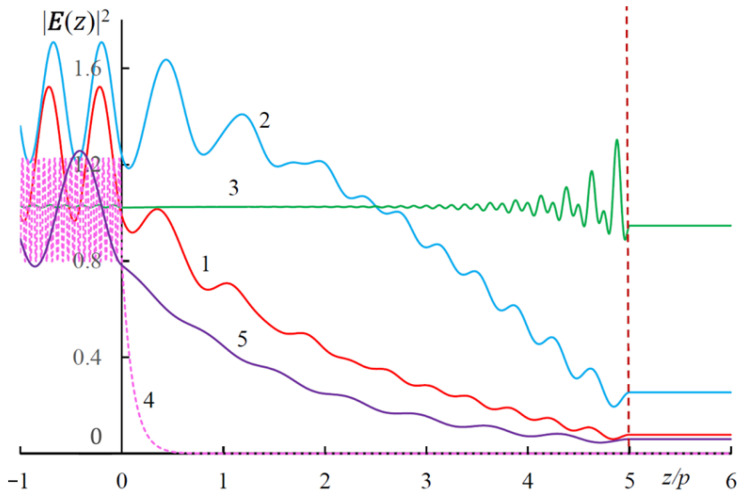
The dependences ${\left|E\left(z\right)\right|}^{2}$ for the following characteristic wavelengths: (1) $\lambda_{0}$ = 282.8 nm; (2) $\lambda_{r}$ = 267 nm; (3) $\lambda_{t}$ = 89.4 nm; (4) λ = 30 nm << $\lambda_{0}$; (5) λ = 500 nm >> $\lambda_{0}$.

Now we will study the features of light localization in the dichroic layer of CLC. We show the total electric field for three distinct cases, namely for the right and left outer sides of the dichroic CLC, and for the CLC layer itself. The CLC borders two isotropic half-spaces, *z* = 0 and *z* = *d*. The total electric field is represented as follows:

(6) $$E\left(z\right)=\{\begin{array}{c} E_{i}\left(z\right)+E_{r}\left(z\right),\;z<0,\\ E_{in}\left(z\right),\;0<z<d,\\ E_{t}\left(z\right),\;z>d, \end{array}$$

where $E_{in}\left(z\right)$ is the total electric field in the CLC layer. Figure 10 shows the distribution of ${\left|E\left(z\right)\right|}^{2}$ at the following characteristic wavelengths at g = 0.3:

1. the wavelength $\lambda_{0}$ of the diffraction reflection in the absence of external magnetic field,
2. the wavelength $\lambda_{r}$ of the diffraction reflection in the presence of external magnetic field,
3. the wavelength $\lambda_{t}$ of the magnetically induced transparency,
4. the wavelength much smaller than the wavelength $\lambda_{0}$,
5. the wavelength much longer than the wavelength $\lambda_{0}$.

Now we turn to investigation of the peculiarities of the light energy density. In this layer, the light energy density is calculated as:

(7) $$w=\frac{1}{d}\int_{0}^{d}{\left|E_{in}\left(z\right)\right|}^{2}dz$$

**Figure 11:**
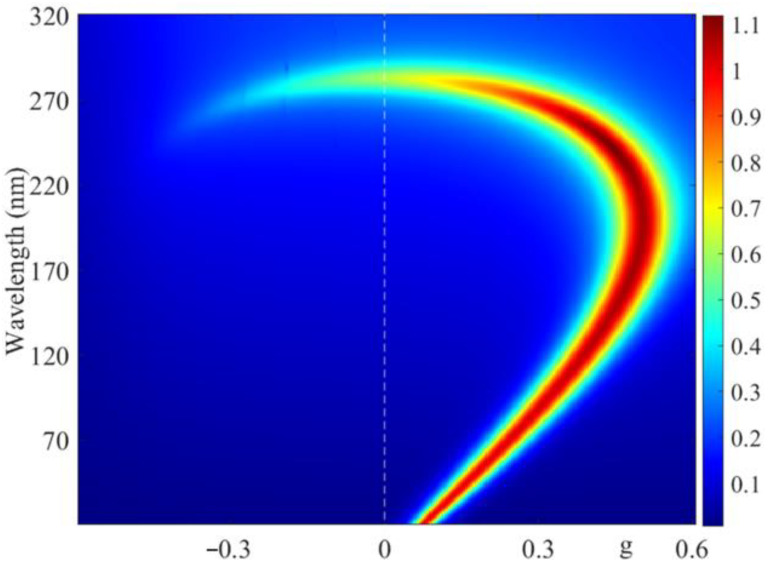
The evolution of light energy density spectra w in the dichroic CLC layer with a change in g. The incident light has diffracting EP.

Figure 11 shows the evolution of light energy density spectra w in the dichroic CLC layer with a change in g. As shown in this figure, a comparatively large light energy accumulation takes place at the wavelengths of magnetically induced transparency and diffraction reflection. It is worth reminding that the medium is absorbing.

**Figure 12:**
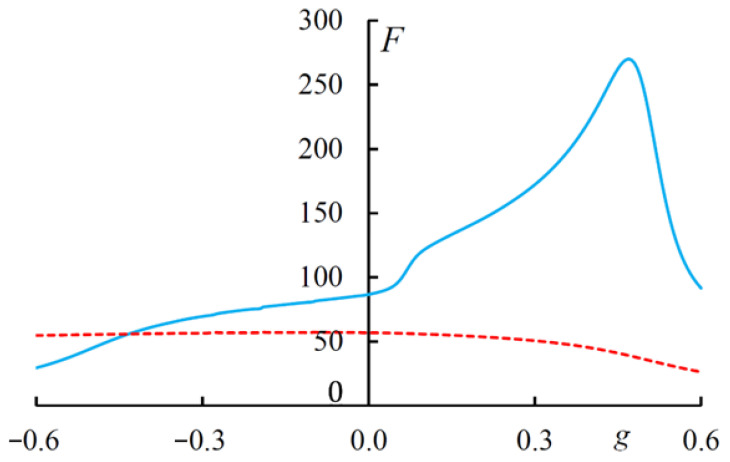
The light energy *F* versus parameter g. The incident light has a diffracting (solid line) and non-diffracting (dashed line) EP. $\lambda_{1}=20$ nm and $\lambda_{2}=320$ nm.

Finally, we calculate the total light energy *F* localized in the dichroic CLC layer in the presence of an external magnetic field in a finite spectral range using the following formula:

(8) $$F=\int_{\lambda_{1}}^{\lambda_{2}}wd\lambda .$$

and evaluate its dependence on parameter g in Figure 12. As is seen in Figure 12, at the change of parameter g in the interval from −0.6 to 0.6 *F* changes in a large interval. *F* has its main maximum at the value g at which the magnetically induced transparency band and the diffraction transmission band merge into a single band. Thus, the change in parameter g allows to substantially control the total light energy localized in the dichroic CLC layer, in addition, this energy has a nonreciprocal property.

**Figure 13:**
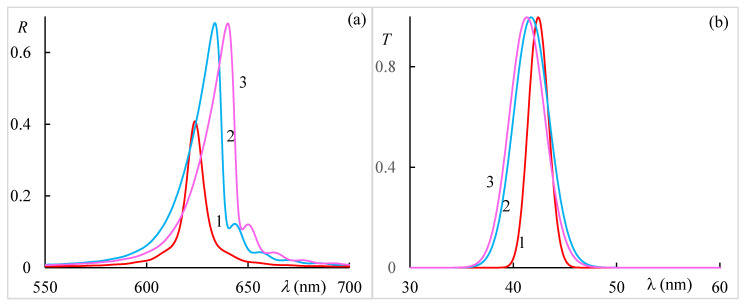
(**a**) The spectra of reflection near the PBG and (**b**) the spectra of transmission near the magnetically induced transparency.

In the last part of this paper, toward more realistic implementations of magneto-active CLCs, we consider that the CLC is endowed by a local dielectric anisotropy. According to Mayer [38], the influence of an external magnetic field on the helix pitch in the lowest order approximation is defined as:

(9) $$p\left(H\right)=p\left[1+\frac{\chi_{a}^{2}H^{4}p^{4}}{32{\left(2\pi \right)}^{4}k_{22}^{2}}+\ldots \right],$$

where $x_{a}$ is the anisotropy of magnetic susceptibility, $k_{22}$ is the coefficient of elasticity. Since in our case $\hat{\mu }=\hat{I}$, we can neglect the helix pitch change in the lowest order approximation. Furthermore, as mentioned above, the magnetic field can also directly affect the local dielectric tensor components, which are a quadratic in the external field effect. Therefore, according to [34], we can present the principal values of dielectric tensor components in the form of:

(10) $$\varepsilon_{1,2}\left(H\right)=\varepsilon_{1,2}+\alpha_{1,2}g^{2},$$

where $\alpha_{1,2}$ are some coefficients of proportionality. Figure 13 shows (a) the spectra of reflection near the PBG and (b) the spectra of transmission near the magnetically induced transparency. The simulation is made for a CLC with the parameters $\varepsilon_{1,2}=2.29$, $\varepsilon_{2}=2.143+0.1i$, p=420 nm. The CLC layer thickness is *d* = 25*p* and magneto-optical activity parameter is g = 0.3. The following three cases are considered in our calculations, namely, (1) the CLC without local birefringence with Reε1=Reε2=Reεm=2.2165, (2) the CLC with local birefringence with $\alpha_{1}=\alpha_{2}=0$, and finally (3) the CLC with local birefringence but with $\alpha_{1}=0.51$, and $\alpha_{2}=0.49$. As is seen from Figure 13, the presence of a local birefringence causes the formation of PBG of a finite frequency width. The difference of $\alpha_{1,2}$ from zero brings the displacements of both PBG and magnetically induced transparency bands. Moreover, if the PBG has a red shift, then the magnetically induced transparency band has a blue shift. At the same time, this modeling shows that the main results and conclusions presented above depend on the local anisotropy, but not crucially, and they remain valid. Taking these effects into account leads to quantitative, but not qualitative, effects. Alternatively, the current pace of development of science and technology suggests that the environments discussed above can be created artificially. On the other hand, it is important to note, that the results obtained above make it possible to construct more accurate magneto-optics for CLCs.

## 4. Conclusions

In conclusion, the magneto-optical properties of a dichroic CLC layer at large values of magneto-optical parameter g and low values of dielectric permittivity $\varepsilon_{0}$ were investigated. We studied the solutions of the dispersion equation and their peculiarities in detail. In [33], the specific properties of solutions of the dispersion equation of conventional CLCs with local birefringence were investigated, and it was shown that the external magnetic field causes displacement of the real parts of the wave vectors parallel to axis *k* = 0. As our investigation shows, an external magnetic field can cause, not only displacement of the real parts of the wave vectors, but also displacement of their imaginary parts. Moreover, it was demonstrated that, at some values of the magneto-optical parameter in the short-wavelength part of the spectrum, a transparency band appears with Im*k_m_* = 0. This band moves to long waves with the increase of parameter g. At further increases in parameter g, this band merges with the diffraction transmission band. The solution to the boundary value problem really shows the existence of a band with *T* = 1 at *A* = 0 and *R* = 0. Thus, we have a magnetically induced transmission band in the absorbing medium. This band is nonreciprocal and tunable. The specific properties of reflection, transmission, absorption, rotation, ellipticity spectra, and also the spectra of ellipticity and azimuth of eigen polarization were investigated as well. Finally, we investigated the specific properties of light localization and integral light localization for this system in the situation of the formation of both the band of diffraction transmission and band of magnetically induced transmission. In [39] an EIT interaction was demonstrated in silicon nanosphere oligomers, wherein the strong magnetic resonance couples with the electric gap mode effectively to markedly suppress the reflection. As a result, a narrow-band transparency window is created at visible wavelengths, called MIT. In [40] similar results were obtained for amorphous silicon nano-disks covered with a magnetic surrounding. In our opinion this effect can also be termed MIT, because it arises in absorbing media due to an external magnetic field and the wavelength of arisen band of full transmission can be tuned by this field. Let us note that nonreciprocal systems, not only allow the development of key photonic components, such as optical isolators and circulators on a chip, but also provide novel ways to transport and process data in photonic systems.

## Data Availability

Data sharing is not applicable to this article.
